# What are the unintended patient safety consequences of healthcare technologies? A qualitative study among patients, carers and healthcare providers

**DOI:** 10.1136/bmjopen-2024-089026

**Published:** 2024-11-27

**Authors:** Shahd Abdelaziz, Sara Garfield, Ana Luisa Neves, Jill Lloyd, John Norton, Jackie van Dael, Carly Wheeler, Monsey McLeod, Bryony Dean Franklin

**Affiliations:** 1 Pharmacy, Imperial College Healthcare NHS Trust, London, UK; 2 UCL School of Pharmacy, London, UK; 3 Department of Primary Care and Public Health, Imperial College London, London, UK; 4 Centre for Medication Safety and Service Quality, Imperial College Healthcare NHS Trust, London, UK; 5 Patient Safety Translational Research Centre, Imperial College, London, UK; 6 Nuffield Department of Primary Care Health Sciences, University of Oxford, Oxford, UK; 7 NHS England, London, UK

**Keywords:** QUALITATIVE RESEARCH, Health informatics, Telemedicine, Information technology, eHealth

## Abstract

**Objective:**

To identify patient-safety-related unintended consequences of healthcare technologies experienced by their primary users: patients, carers and healthcare providers (HCPs).

**Design:**

Qualitative study based on data collected in online focus groups. Transcripts were analysed inductively after each focus group using reflexive thematic analysis, focusing on identifying unintended consequences of healthcare technologies with implications for patient safety. Patient safety was broadly conceptualised to include a more subjective concept of ‘feeling safe’ as well as risks of actual harm.

**Setting:**

Patient/public and HCP participants from the UK with experience in healthcare technologies were recruited using a mixture of purposive, convenience and snowball sampling.

**Participants:**

40 participants (29 patients/public, 11 HCPs) took part in 5 focus groups between November 2021 and February 2022.

**Results:**

We identified five main themes of unintended consequences with implications for patient safety: inequity of access, increased end-user burden, loss of the human element of healthcare, over-reliance on technology and unclear responsibilities. Both groups of participants identified unintended consequences directly affecting patients; HCPs also described those affecting themselves. Some unintended consequences are described in previous literature, including alert fatigue, the ‘illusion of communication’, reduced opportunities for face-to-face interactions and increased end-user burden. Others are potentially novel, including patients’ psychological dependence on technologies, ‘gaming’ of data entry and incorrect interpretation of health data.

**Conclusions:**

Drawing on the perspectives of patients/public as well as HCPs, we identified five areas of patient-safety-related unintended consequences associated with healthcare technologies. These should be considered when developing tools to identify and mitigate the patient-safety-related unintended consequences of healthcare technologies.

**STRENGTHS AND LIMITATIONS OF THIS STUDY**

- A strength is that we included a diverse group of patients/public as well as front-line healthcare professionals from a broad range of organisations.
- A limitation is that participants were from one country and all spoke English which may limit generalisability to different contexts, cultures and practices.
- It may have been difficult for participants to identify whether patient safety issues were consequences of technology rather than broader issues within healthcare such as geographical variation in services and insufficient resources.
- Data were collected prior to the more widespread availability of generative artificial intelligence, which may have affected the findings.

## Introduction

 Healthcare organisations often introduce new technologies aimed at supporting patient safety or other aspects of healthcare quality. These include electronic prescribing systems,^1^ ‘smart’ intravenous pumps,^2^ decision support systems to aid information provision and decision-making^3^ and home diagnostic tests.^4^ While such technologies have significant potential benefits, they can also introduce new patient safety risks^5^,^10^ a concept sometimes referred to as e-iatrogenesis for digital technologies.^11^ They may also have other unintended consequences, such as reducing patients’ opportunities for engaging with their healthcare.^12^ This is partly because technologies used in healthcare do not operate in isolation but form part of a broad sociotechnical system comprising numerous inter-related technologies and human factors.^13^,^18^ Identifying and addressing any negative unintended consequences is, therefore, important to maximise the potential benefits of such technologies.

Existing tools and frameworks to support use of technologies in healthcare^19^,^22^ tend to focus on high-level organisational issues around implementation, rather than identifying and mitigating any negative unintended consequences that may arise once technologies are in use. They also focus on considerations for managerial and non-patient-facing staff, rather than the perspectives of those who directly use the technologies: patients, carers and front-line healthcare providers (HCPs). Furthermore, many existing frameworks are specific to one area of technology, for example, electronic health records^23^ or electronic prescribing.^21^ There are likely to be common considerations across technologies, which could be potentially used to develop a more generic framework with wider potential benefit. Importantly, there has also been little, if any, input from patients, carers and members of the public into existing tools and frameworks.^19^,^23^ As patients and their carers are at the centre of care, they have the potential to highlight important issues that may be different to those identified by HCPs.

In this study, we therefore aimed to explore the patient safety-related unintended consequences of healthcare technologies, and how these might be mitigated, from the perspectives of their primary users: patients, carers and patient-facing HCPs.

## Methods

### Study design and research philosophy

We conducted a qualitative study based on online focus groups with patient/public and HCP participants from the UK. We adopted a critical realist epistemology and an interpretivist methodology, drawing on participants’ subjective perspectives and experiences to identify and describe unintended consequences. Reporting adheres to the Consolidated Criteria for Reporting Qualitative Research.^24^

### Selection and recruitment of participants

Adult patient/public participants with experience in healthcare and who could communicate in English were recruited through our research networks plus an online external research–participant organisation.^25^ The latter provides access to a diverse range of public participants who have signed up to receive information on research opportunities. We invited expressions of interest from those who met our inclusion criteria and then selected participants using purposive sampling to support diversity in age, gender and ethnicity. HCPs were recruited via email, using a combination of convenience and purposive sampling via the research team’s networks, followed by snowball sampling, aiming for diversity in roles and backgrounds.

Potential participants were emailed by the research team, including an information sheet and invited to respond if they wished to take part. Participants were asked to provide written informed consent; patient/public participants were offered an honorarium of £75.^26^

Prior to the focus groups, topic guides were piloted among the research team. In line with an interpretivist approach, transcripts were not returned to participants for accuracy checking or to provide feedback on the findings.

### Identifying technology-associated patient safety risks

We conducted focus group discussions using a video conferencing platform (Zoom, with dial-in option), between November 2021 and February 2022. Three members of the research team (one public partner and two researchers) facilitated each focus group. The researchers comprised pharmacists, GPs and health services researchers and had experience in facilitating online discussions.

A short presentation was delivered at the start of the discussion outlining the study’s aim and providing examples of technologies used in healthcare (patients’ online access to healthcare records, electronic prescribing systems and point-of-care blood glucose testing). Technologies were deliberately broadly framed as any tool that patients, carers and/or clinicians use to help manage a patient’s health, illness or information about their care. Participants were then invited to discuss (1) examples of healthcare technologies they used or knew of; (2) potential unintended consequences of such healthcare technologies on patient safety and (3) potential ways to identify and mitigate these (online supplemental appendix 1).

Participants contributed to the discussions by verbal and/or written communication using the Zoom chat function or via Padlet, an online noticeboard platform. Facilitators encouraged all participants to contribute. Audio and written discussions were recorded with the former transcribed verbatim; any participant identifiers were removed.

Reflexive thematic analysis was used, focusing on identifying unintended consequences with potential implications for patient safety, conceptualised as outcomes not originally intended that could affect patient safety, either directly or indirectly. Patient safety was broadly conceptualised to include a more subjective concept of ‘feeling safe’ as well as actual or potential harm. Preliminary analysis took place after each focus group followed by a more comprehensive analysis once all had been completed. Data were coded inductively by two pharmacist researchers (SA and SG, using NVivo V.R1.6.1) and two lay partners (JL and JN, using manual coding) in an iterative process to develop initial themes and subthemes. Different perspectives were addressed through discussion to reach a deeper understanding of the data and its interpretation. The main overarching themes and subthemes were developed through further iterative analysis led by SA and BDF and then reviewed by all authors. Focus groups were conducted until thematic saturation was achieved, defined as when no new relevant themes were identified during the preliminary analysis that took place after each focus group.^27^

### Patient and public involvement

Public partners, JL and JN, were involved in developing the topic guides, facilitating focus groups and data analysis. They suggested conducting the final two focus groups solely with patient/public participants to gain a wider range of perspectives from this group. The initial topic guide was also altered to explore hypothetical unintended consequences, in addition to real experiences, in response to JN’s suggestions.

## Results

40 participants (29 patients/public, 11 HCPs) took part (table 1) in 5 focus groups each of 1.5 hours’ duration. The first three comprised a mixture of patient/public and HCP participants; the remaining two comprised solely patients/public. Two patient/public participants were already known to the research team. Four additional patients/public and two HCP participants who consented to take part did not subsequently attend, with no reason provided. A further patient/public participant withdrew before the focus group due to personal circumstances. Box 1 gives examples of the range of technologies discussed.

**Table 1:** Participant self-reported demographic characteristics

	Total patient/public participants	Doctor	Nurse	Pharmacist	Total healthcare provider participants
Gender					
Female	20	1	1	2	4
Male	9	3	0	1	4
Not specified	0	2	1	0	3
Total	29	6	2	3	11
Age					
20–29 years	7	0	0	0	0
30–39 years	0	2	0	1	3
40–49 years	6	2	1	1	4
50–59 years	7	0	0	0	0
≥60 years	6	0	0	0	0
Not specified	3	2	1	1	4
Total	29	6	2	3	11
Ethnicity					
African-Caribbean/ British African/ Caribbean	2		1		1
Asian/British Asian	9	1		1	2
Hispanic				1	1
Mixed	1	1			1
White British	11	1			1
White European	1	1			1
Not specified	5	2	1	1	4
Total	29	6	2	3	11

Box 1 **Examples of healthcare technologies discussed by participants**

Online appointment booking and consultation platforms.

Clinical decision support systems.

Mobile health applications.

Fitness trackers.

Smart drug delivery systems and implants, for example, insulin patches.

Technologies that use machine learning and artificial intelligence.

Robotics.

Patient medical record platforms for example, electronic health records.

Patient home diagnostic and monitoring devices, for example, blood pressure machines.

Remote consultation technologies, for example, video conferencing platforms.

We identified five main themes of unintended consequences with implications for patient safety (table 2). Most consequences identified by patient/public participants were similar to those described by HCPs, although the former focused on technologies used by patients directly. HCPs also described further unintended consequences associated with technologies designed for HCPs’ use.

**Table 2:** The five themes, and associated subthemes, for unintended consequences of healthcare technologies on patient safety

Key themes	Potential patient safety consequences	Subthemes
Theme 1: inequity of access	Reduced access to health services	Disparities in use and access to healthcare technologies due to: Physical diversities and neurodiversities Digital poverty Low digital literacy ‘Postcode lotteries’ in healthcare services
Theme 2: increased end-user burden	Reduced psychological safety, alert overload and/or increased opportunity for error	Additional patient tasks and psychological burden Increase in patient anxiety Due to feeling the need to constantly check that the technology is working Due to misinterpreting or overthinking their health data Increased healthcare professional burden due to multiple log-ins and alert fatigue and/or having to input patient health data into multiple systems Lack of interoperability between different technologies resulting in fragmentation of patient health data
Theme 3: loss of human element of healthcare	Reduced psychological safety and/or potential increase in diagnostic error	Reduced in-person interactions Increased social isolation for patients Increased risk of missed symptoms, incorrect diagnoses and delays in accessing healthcare Potentially receiving distressing health-related information without in-person support
Theme 4: over-reliance on technology	Reduced psychological safety and/or reduction in backup systems and safeguards leading to mismanagement of healthcare conditions	Patients develop psychological dependence on technologies, then feel ‘a sense of abandonment’ when these are no longer available ‘Illusion of communication’ Patients perceive that information they enter into patient-held technologies will be seen and acted on by healthcare professionals, when this may not always be the case Healthcare professionals perceive that they will be alerted by the technologies if patients’ health information displayed to them is incorrect when this may not always be the case Assumptions that technologies will always work as expected, which limits provision of back-up systems for when they fail Healthcare professionals’ trust in information generated by and/or displayed to technologies, when this may not always be appropriate
Theme 5: unclear responsibilities	Reduced psychological safety	Unclear responsibilities if health deteriorates Unclear responsibilities when things go wrong Unclear responsibilities surrounding the security of stored data

### Inequity of access

Participants highlighted disparities in use and access, such as how physical diversities and neurodiversities, digital poverty and low digital literacy can result in patients not being able to use or access healthcare technologies, with downstream implications for patient safety. Patient/public participants, in particular, highlighted these problems, and felt that these issues are often overlooked by HCPs.

I think a lot of the time, technology fails to be adaptive for different people, whether that’s physical challenges or to do with different kinds of visual impairments, or learning differences, and autism spectrum disorder. Patient/public, focus group (FG)5

I think there’s an assumption that we all have broadband and we all have technology but actually, digital poverty’s also a big one. Patient/public, FG4

One participant highlighted how patients who cannot access or use technologies because of such barriers may then experience reduced access to healthcare.

They might think, well, I can’t go online, therefore, I’m not going to communicate my problem. Patient/public, FG2

In contrast, however, some participants highlighted how technologies can also make it more convenient for patients to access HCPs, and that some patients prefer remote consultations, thus increasing access.

If we’re thinking about things like remote consultations and virtual clinics […] that might allow more people to attend their meetings and consultations. HCP, FG1

Last time I spoke to my doctor and she was saying that a lot of the younger generation now are saying they far prefer telephone calls. Patient/public, FG1

Finally, participants suggested that different patients may be provided with the same type of technology, but of varying quality, depending on where they live or which provider is managing their health. This could lead to further health inequities.

My cousin and I had diabetes at the same time and she was under an endocrinology department and she got a very sturdy piece of equipment to do her bloods. And I got this tiny little thing…it was very fragile. Patient/public, FG4.

### Increased end-user burden

Participants explained how using healthcare technologies can add to the psychological and/or physical burden experienced by patients in managing their health, leading to concerns about safety. An example was how patients experience the pressure of needing to know how to use technologies correctly, particularly where they are concerned about the potential consequences of erroneous data being entered or generated.

If you don’t know what you’re doing with the technology, digital or otherwise, then you may not know whether you’re doing the right thing […]. HCP, FG1

I can see possibilities of it being extremely stressful, for me, it felt like an exam that I needed, everything depended on me passing that day […] because of the nature of preeclampsia so it was exceptionally difficult. Patient/public, FG4

Participants also highlighted how some patients do not know who to contact when they have difficulties using technologies, or that support services are increasingly online which can create a barrier to access, resulting in patients being unable to manage their health as intended.

They had no details to call the provider to get a new code to log in, now why would you even embed such a complicated system into giving the patients an app system to use. HCP, FG2.

Patient/public participants described increased anxiety when using technologies. They explained how they may constantly check that technologies are working, or feel driven to keep checking their own health data. This could lead patients to ‘overthink’ their health conditions, particularly if they misinterpreted their results.

The fact that she had this thing [technology] that was meant to simplify her life actually made it more complex because she was thinking that it either didn’t work, or she had to contact someone […] it was an infernal loop. Patient/public, FG4

If you have a patient-held app […] sometimes you have results and things which are abnormal based on standard values but for your clinical condition […] it may be less abnormal or it’s not a concern […] it may harm the patient’s mental wellbeing. HCP, FG1

User fatigue was a key concern for HCPs, who often had to log in to numerous systems and receive a high volume of (often irrelevant) alerts, prompting them to dismiss them and forget to complete other tasks.

I think sometimes it can be too much and I think it’s really easy for users to just press accept or ignore and then sometimes there is a risk that we ignore information that they should be acknowledging. HCP, FG3

HCPs conveyed frustrations surrounding the resulting inefficiencies and duplications, resulting in greater workload with opportunity costs elsewhere, as well as negative emotions affecting patient care.

There may be potentially a duplication of work rather than a saving of time if, for example, information needs to be entered twice into one system and then into another system …. HCP, FG1

A burden shared by patients/public and HCPs was lack of interoperability. Participants described how different healthcare services, and even different teams within the same organisation, use different technologies that are not well integrated. For patients, this resulted in having to continuously update different healthcare teams, or input data on different systems, increasing opportunity for error. They also reported receiving conflicting advice since different teams cannot view what others have advised. HCPs reported how lack of interoperability caused them to miss important information.

It’s an absolute nightmare trying to explain what drugs you’re on to every single clinician you come in contact with, and what your conditions are—it baffles me because it must just be so detrimental to the health service. Patient/public, FG5

As part of data existing in multiple different places where it’s entered on a particular app and then it’s also entered on a clinical record, for example, if those two don’t talk to each other you can end up with inconsistencies and fragmentation of data. HCP, FG1

### Loss of human element of healthcare

Both patient/public and HCP participants highlighted how features of traditional in-person interactions were lost with remote technologies. They discussed concerns surrounding removal of the safeguards of physical examinations, increasing the risk of physical, mental and social symptoms being missed.

I saw someone with some anxiety symptoms and it turned out to have been caused by a physical health problem but this wasn’t apparent on the telephone call. HCP, FG3

People who are vulnerable with mental health needs, people who are even isolated, they might need that [face-to-face] interaction from their doctor because I would say that the doctor provides a social care need. Patient/public, FG5

Another participant explained how lack of in-person interactions meant that health concerns were often not addressed within an appropriate timeframe, due to having to use online systems before gaining direct HCP contact.

Family members have had to go back into the NHS 111 app, and then you book it, and you get through to your [primary care] practice, and then you send something, and then you get a telephone consultation […] I think basically you don’t get care quick enough at the moment and all we’re doing is leaving care and problems for later. HCP, FG2

Interactions with HCPs via digital technologies were considered less personal than face-to-face interactions, with concerns that this could discourage patients from sharing personal and important health information. A patient/public participant emphasised how potentially distressing information should be given face to face.

I think there are certain parts perhaps patients would be less willing to share and when you’re seeing someone face-to-face it can be easier to share those stories. HCP, FG3

Things like cancer, you wouldn’t want to get a result or something, or a screening done and then get delivered that news by the technology. Patient/public, FG5

### Over-reliance on technology

Participants explained how patients can develop psychological dependence whereby they feel that technologies are integral to managing their health. It could then be distressing if technologies stop working or are considered no longer necessary by HCPs.

My cousin, she had some monitoring equipment that saved her life because her oxygen levels dropped. And then when it was taken away […] she was absolutely devastated but it was only part of a pilot. Patient/public, FG4

HCPs suggested that patients sometimes did not escalate important health concerns as they assumed data input into health technologies was seen and acted on by HCPs, resulting in an ‘illusion of communication’. One patient/public member also questioned whether patients are relying solely on the technology to be alerted of any health concerns, or errors.

There’s potentially a patient safety issue that arises from patients believing that entry will be actioned, particularly if it’s an urgent one when perhaps the pathway’s not built towards that. HCP, FG1

They [patients] won’t take responsibility for aspects of their health because [they think] with the machines that’s dealing with it, I’ll know if there’s a problem. Patient/public, FG4

One participant provided an example of how HCPs may share this belief:

She [a nurse] said ‘somebody must have checked it or the system would tell me if it wasn't right’. HCP, FG1

HCP participants discussed how it is assumed that technologies will always work as intended, with robust back-ups not necessarily in place. Some technologies had also been used for so long that newer staff are unaware of traditional work practices that would serve as back-ups if needed, potentially putting patients at risk.

And we’ve got a cohort of staff who’ve never seen a paper prescription chart and would probably not feel very confident in using that when the time came. HCP, FG1

Both participant groups emphasised concerns at the level with which HCPs trust the information provided, perceiving all information to be correct, which could result in incorrect clinical decisions.

With technology I think people are loathed to even consider that technology could make a mistake. HCP, FG1

Related to this, one HCP suggested patients may also falsify results on digital technologies to increase the perceived severity of their condition and expedite treatment, which could result in patients in genuine need being deprioritised.

Some patients were actually falsifying their results so that we would get a trigger and bring them in earlier. HCP, FG2

Finally, healthcare technologies that use ‘black-box’ systems such as artificial intelligence produce outputs without users necessarily knowing how they were generated. HCPs highlighted how this left them unsure whether they were applicable to specific patients, potentially resulting in wrong diagnoses or treatments. One HCP questioned how safe patients would feel if they were aware of how such systems generate recommendations.

With an algorithm or a machine learning system that’s aggregating data from a large dataset it just gives you the output, the outcomes, and so you don’t really know it’s made that particular decision and whether it’s factoring any of the subtleties. HCP, FG3

We never really understand how they reach their decisions a lot of the time and I guess there’s an issue, how comfortable do people feel with a system coming up with a decision on their healthcare that they don’t know how something arrived to it. HCP, FG3

### Unclear responsibilities

Participants suggested patients may not feel safe when using healthcare technologies due to lack of clarity as to where responsibility lies in specific situations, such as if their health deteriorates or the technology stops working.

If you’re recording that data because the patient’s wearing it, even though they know you’re not watching it that’s still a risk because you’re recording data saying the patient’s unwell and nobody’s doing anything about it. HCP, FG3

One patient/public participant suggested that due to absence of defined responsibilities, such issues may be recognised and addressed only after a safety incident.

What if things do go wrong, who is responsible, is it the person on the company who designed it, is it the manufacturer of a device, is it maybe a pharmacist, a doctor, consultant, no one? So obviously that’s an issue that needs to be thought of because I can’t wait until for something goes wrong for them to say, OK we need to remedy this. Patient/public, FG5

Many participants highlighted a potential security risk for health data stored on digital health technologies, due to concerns about unintentional or malicious leaks, and were concerned about transparency as to who was responsible for data security.

There is a bit of a risk with data in healthcare, just in regards to any device that we have that’s recording data, is going to be vulnerable to somewhere along the chain. HCP, FG3

Others disagreed, perceiving that technologies are more secure than traditional paper-based systems.

### Mitigations

Participants also provided possible mitigations to the unintended consequences discussed (box 2).

Box 2 **Mitigations suggested by focus group participants**

Technology design.

Diverse user engagement and involvement in technology design and implementation.

Develop context-specific strategies to address and/or mitigate challenges in intraoperability with other technologies already in use.

Using technology in clinical practice.

Confirming patients’ accessibility to the technology and provide alternatives to the technology as needed.

Manage patients’ expectations of how the technology will help them.

Offer an option for in-person consultations where relevant.

Ongoing mitigation of patient safety risk.

Actively seek regular user feedback and monitor the technology in use and any reported unintended consequences.

Provide clear guidance for all stakeholders involved over their responsibilities.

Ensure technical support that can be readily and easily accessed by end-users.

Provide training for users on how to use the technology.

## Discussion

### Principal findings

HCP and patient/public participants discussed a range of health technologies; all were relatively ‘high tech’ and/or digital. Participants conceptualised patient safety more broadly than anticipated, leading to identification of five main themes of unintended consequences with implications for patient safety: inequity of access, increased end-user burden, loss of human element of healthcare, over-reliance on technology and unclear responsibilities. Our work builds on the limited literature on patients’ experiences of unintended consequences of healthcare technologies. There was notable overlap in the unintended consequences identified by patient/public and HCP participants. Both groups of participants described direct experiences of unintended consequences. HCP participants also described the unintended consequences they envisaged patients to experience as a result of using technologies. Overall, our findings suggest unintended consequences identified by patients and carers are similar to those identified by healthcare professionals both in our study and in previous literature.

### Comparison with previous literature

Our findings support those of others suggesting that technology can create what some refer to as a ‘digital divide’^18 19^ and others to ‘intervention-generated inequalities’,^28^ exacerbating inequalities in healthcare due to differences in accessibility, use, adherence and/or effectiveness in different socioeconomic groups. Our participants saw this as a patient safety issue and also emphasised further inequalities arising from physical diversity and neurodiversity. Patient safety consequences of remote consultation technologies in terms of the risk of missed diagnoses, the consequences of technology breakdowns, lack of interoperability and appropriate back-ups have also been described elsewhere.^29^,^32^ There was a particular concern among many participants that HCPs perceive the data presented on technologies will always be correct. However, in contrast, we also identified HCP mistrust in ‘black-box’ systems, as recognised previously.^33^

Our findings also build further on previous work. While it has long been recognised that being unwell carries a ‘burden of treatment’,^34^ with some studies suggesting that technology can decrease this burden,^35^ our findings suggest that technology can increase rather than decrease this burden, both for patients and HCPs. Other unintended consequences such as digital exclusion, impersonal interactions, receiving worrying results via technology and incorrect interpretation of health data have been previously identified, adding cumulative validity to these findings.^36 37^ We identified additional risks around patients’ psychological dependence on technologies, ‘gaming’ of data entry and unclear responsibilities for when ‘things go wrong’ that have not previously been highlighted as patient safety risks. Some of the unintended consequences we identified are likely to reflect wider issues in healthcare, such as ‘postcode lotteries’ in healthcare quality.^38 39^ Our findings also highlight how health data presented on digital technologies is not without context and is open to interpretation depending on who is interpreting it and in what situation, with potential consequences for both physical and psychological safety.

The consequences identified include some that may be considered ‘anticipated’ as well as those that are likely ‘unanticipated’.^40^ For example, missed diagnostic opportunities might be an anticipated consequence of remote consultations (termed a ‘trade-off’ by Bloomrosen *et al*^40^), while increased patient anxiety due to feeling the need to regularly check data recorded by a remote monitoring device may be less easy to anticipate. We also suspect that the ability to anticipate such consequences is likely to change over time and context.

We identified examples of consequences in the first four of the five types of interaction described in Harrison *et al*’s Interactive Sociotechnical Analysis framework^18^ namely new technology changing existing social systems, technical and physical infrastructures mediating technology use, social systems mediating technology use and technology-in-use changing social systems. We did not identify examples of the final type of interaction, in which technology–social system interactions engender technology redesign, although this may reflect our study design.

### Strengths and weaknesses

Strengths include the inclusion of a diverse group of patients/public as well as front-line HCPs from a broad range of organisations, and the inclusion of public partners in design, data collection and analysis. However, participants were from one country and all spoke English which may limit generalisability; unintended consequences associated with technologies with which our respondents were unfamiliar, such as systems used in the USA,^41^ may not be represented. About two-thirds of patient/public participants were female; the experiences of male patients may, therefore, be under-represented. We did not record participants’ geographical areas, although the researchers noted that both HCPs and patients/public represented different parts of the UK. Other limitations include our findings being based only on participant perceptions; interestingly, we noted that HCPs often commented on patients’ views and vice versa. In some cases, it may have been difficult for participants to identify whether issues were consequences of technology rather than broader issues within healthcare such as geographical variation in services and insufficient resources. Data were also collected prior to the more widespread availability of generative artificial intelligence which may account for this not being discussed.

### Implications for clinicians and policy-makers

We have identified five themes of unintended consequences of healthcare technologies that potentially pose patient safety risks. These should be considered when developing tools to identify and mitigate the patient-safety-related unintended consequences of healthcare technologies. As recently highlighted, ‘innovations are rarely developed with inclusive testing of patient safety as part of the initial design process’^42^; our participants’ suggested mitigations also emphasised the importance of diverse user engagement and involvement in technology design.^43^ Our work complements existing frameworks of risks associated with specific technologies such as remote consultations^44^ and hospital information systems.^45^

### Implications for research

Further research should include observation of technology in practice to provide additional insights around sociotechnical interactions and the broader context in which technologies are used, allowing for triangulation of observational data with user perspectives. Further exploration is also needed in relation to whether unintended consequences are considered positive or negative, and how this may differ between stakeholder groups and/or change between different settings and contexts of use.^9^ For example, our findings suggest that patients’ ‘gaming’ of data was viewed as a patient safety risk, but such ‘workarounds’ can also establish safety in other situations if used to correct for systems that are not working appropriately.^44^ Future research could explore the perspectives of technology developers on how to respond to the unintended consequences identified.

## Conclusion

Based on input from a wide range of HCPs and patients/public, we identified five areas of unintended patient safety consequences associated with use of technology in healthcare. Our findings could be used to develop tools to aid in design and implementation of healthcare technologies, in order to identify and mitigate these unintended consequences. Our findings also highlight the importance of sufficiently diverse user engagement and involvement in technology design and implementation.

## supplementary material

10.1136/bmjopen-2024-089026 online supplemental file 1

## Data Availability

All data relevant to the study are included in the article or uploaded as supplementary information.
