# Supplementary material for: What are the unintended patient safety consequences of healthcare technologies? A qualitative study among patients, carers and healthcare providers
Source: BMJ Open. 2024 Nov 27;14(11):e089026. doi: 10.1136/bmjopen-2024-089026 (PMC11603710; doi:10.1136/bmjopen-2024-089026)
Supplement: online supplemental file 1 [file bmjopen-14-11-s001.docx]

**Appendix 1**

Safety and Unintended Consequences of Healthcare Technology

Topic guide for focus groups

**Slides to prepare**

- Ground rules
- Definitions
- Case studies

**Facilitator roles**

Facilitator 1: Read the questions, chair the discussion, and monitor hands up (either using zoom functionality or video). Ensure anyone joining by phone has opportunity to contribute to all discussions.

Facilitator 2: Manage the technology e.g. admit people from waiting room, help with any technological difficulties, share and add comments to whiteboard or Powerpoint during part 3. Also monitor chat and input comments from there into discussion (using hands up function so that facilitator 1 can see when to invite incorporation of chat into the discussion). Facilitator 2 may need to join from two devices – one to record, and one to be able to type into and share documents.

Facilitator 3: Take notes of overall impressions of the group and themes emerging from the discussions. Assess accessibility and inclusivity of all participants and any adjustments that may need to be made to increase these for future groups.

**Instructions for the researcher (5 minutes recap as will already have received info and consented)**

Confirm that the interviewees understand:

- The purpose of the research
- What the focus group or interview entails
- How confidentiality and anonymity will be assured
- That they can stop at any time without explanation

And that:

- They have had the chance to ask questions
- They are content to be recorded and that they have the option to switch off their video and use only first names on their Zoom square if they wish

**Topic areas for the focus group**

**Allow 5 minutes for participants to join then start promptly after that.**

***Part 1: Introductions to each other, and to the focus group and topic area (15 minutes)***

1. Introductions, Ground rules. Explain how to use the hands up and chat functions in Zoom. (screen share on)
2. Introductions (screen share off)
3. Definitions as per our study. (screen share on)
4. Introduce case study examples of the introduction of technologies in healthcare settings and examples of unintended consequences (screen share on)
5. Handover to facilitator 2 to begin recording

***Part 2: Use of technologies: (10 minutes)***

1. What other health-related technologies can you think of that you use, or know of?

***Part 3: Identifying unintended consequences (30 minutes)***

1. What types of things may happen when these technologies are introduced that we might not have thought of beforehand?

**Prompt – focus on patient safety**

**How people make decisions**

**The way care is delivered**

**Feel free to give examples from your own experience**

Put these on whiteboard or share Powerpoint and facilitate a discussion re grouping them. Participants can put in the chat for facilitator 2 to add or hands up and speak. NB if anyone is using the video for hands up rather than the zoom hand up function, pin them to your screen so that you can see them during screenshare

***Part 4: Mitigating against negative unintended consequences (15 minutes)***

1. How might we predict negative unintended consequences in advance?
2. What might we do to prevent these consequences?

**Prompts:** Who should be responsible for this?

What would be the best time to do this in relation to introducing the technology?

***Part five: Wrap up (10 minutes)***

1. Is there anything else anyone would like to add?
2. Summary, wrap up and next steps – invitation to next focus group? PPI reimbursement, feedback for future groups, feel free to add any thoughts by email

XX and XXX 03.11.21
