# Supplementary material for: What are the unintended patient safety consequences of healthcare technologies? A qualitative study among patients, carers and healthcare providers
Source: BMJ Open. 2024 Nov 27;14(11):e089026. doi: 10.1136/bmjopen-2024-089026 (PMC11603710; doi:10.1136/bmjopen-2024-089026)
Supplement: Supplementary file 2 [file bmjopen-2024-089026.reviewer_comments.pdf]

PEER REVIEW HISTORY

BMJ Open publishes all reviews undertaken for accepted manuscripts. Reviewers are asked to complete a checklist review form and are provided with free text boxes to elaborate on their assessment. These free text comments are reproduced below.

This paper was submitted to another journal from BMJ but declined for publication following peer review. The authors addressed the reviewers' comments and submitted the revised paper to BMJ Open. The paper was subsequently accepted for publication at BMJ Open.

ARTICLE DETAILS

Title (Provisional)

What are the unintended patient safety consequences of healthcare technologies? A qualitative study among patients, carers and healthcare providers

Authors

Abdelaziz, Shahd; Garfield, Sara; Neves, Ana Luisa; Lloyd, Jill; Norton, John; van Dael, Jackie; Wheeler, Carly; McLeod, Monsey; Franklin, Bryony

---

VERSION 1 - REVIEW

**Reviewer**	**1**
**Name**	**Sittig, Dean**
**Affiliation**	**UTHealth Houston**
**Date**	**14-Mar-2024**
**COI**	**None**

The authors report on results of a small qualitative study involving 29 patients and 11 healthcare providers. It is not clear what questions or prompts were given to participants in the focus groups. If it was just to discuss potential unintended consequences of technology, that doesn't seem realistic. Not sure what technology tools as “digital or non-digital” means? I'm not aware of any analog computers used in healthcare these days, or does this mean something else like non-computer-based technology? Like what? While I like the idea of asking about unintended consequences, several of the unintended consequences identified were pre-defined goals of the project. For example, the general idea of patient-facing technology is to reduce the workload of healthcare providers by letting the patients do things for themselves. Also, the idea was to give patients work to do. Perhaps the patients in

your study didn't want this additional work, but other patients are clamoring for it. Similarly with access to information. While you found that patients didn't want to receive bad news via technology, at least in the USA, our patient representatives in Congress have passed laws requiring healthcare organizations to do just that. This is a trade-off that was not unanticipated or unintended. Also, in this day in age, it is unrealistic for anyone to believe that technology can NOT fail. I mean, who among us hasn't had a computer, phone, Fitbit, watch, or app on their phone fail to work properly? It seems to me that everything fails, often more than it succeeds.

While I appreciate the work that went into collecting and analyzing this data, I'm a bit surprised that more attention was not given to highlighting the differences or similarities between this work and other work focused on unintended consequences of use of health IT (e.g., doi: 10.37765/ajmc.2023.89367; doi: 10.1016/j.apnr.2023.151724; doi: 10.15265/IY-2016-023; doi: 10.1016/j.ijmedinf.2022.104958; among many others). In my opinion, it is important to place your work in the proper context within the existing scientific literature. Your study is not totally unique, I think a good review of the existing literature would help you place your findings in the proper perspective and really help highlight what is new and what is confirmatory in your work.

I found the information in box 2 to be very important, yet it is presented as an unorganized list of notes. I think a more in-depth description of what organizations and Health IT developers can do to help prevent or at least mitigate the problems you have identified is not only justified, but necessary.

I'd also like to see the authors try to map their work to some sort of framework, model, or theory that could help others to place their findings in context. As it now stands, I see 5 unrelated "themes" that make me wonder whether they are comprehensive, or based on the limited sample that was involved in this study.

**Reviewer**	**2**
**Name**	**Reviewer 2**
**Affiliation**	
**Date**	**14-Mar-2024**
**COI**	**n/a**

Comments not published pending reviewer's consent

VERSION 1 - AUTHOR RESPONSE

Reviewers' Comments to the Author	RESPONSES
**Reviewer 1**	**RESPONSES**
Not sure what technology tools as "digital or non-digital" means?	We have now replaced this statement in the paper by giving the specific examples used.
Also, the idea was to give patients work to do. Perhaps the patients in your study didn't want this additional work, but other patients are clamoring for it. Similarly with access to information. While you found that patients didn't want to receive bad news via technology, at least in the USA, our patient representatives in Congress have passed laws requiring healthcare organizations to do just that. This is a trade-off that was not unanticipated or unintended.	We have specified in the study's limitations that the participants were from one country, limiting the generalisability of the results in relation to different contexts.
I'm a bit surprised that more attention was not given to highlighting the differences or similarities between this work and other work focused on unintended consequences of use of health IT (e.g., doi: 10.37765/ajmc.2023.89367; doi: 10.1016/j.apnr.2023.151724; doi: 10.15265/IY-2016-023; doi: 10.1016/j.ijmedinf.2022.104958; among many others	We thank the reviewer for this suggestion. We appreciate that existing literature and frameworks tend to be focused on one type of technology, which is something we highlighted in the introduction. We had framed the discussion to highlight comparisons of common considerations/unintended consequences we identified in our study with those that are in literature. This reflects how we grouped the themes of unintended consequences in the results sections. We've also added more specific comparison with another typology, highlighting the novel contribution of our work.<br><br>The paper by Leuchter and Sarkisian (doi: 10.37765/ajmc.2023.89367) is surrounding physician pay-for-performance programmes, which are unrelated to our study. We had already included the reference by Sittig et al (DOI: 10.15265/IY-2016-023), and feel that we have included several other relevant citations to support our discussion.<br><br>The paper by Rohani and Yusof (doi: 10.1016/j.ijmedinf.2022.104958) is surrounding pharmacy information systems, a technology not used in UK healthcare settings. We have added this paper in the study's strengths and limitations section where we highlight the limitation of our work being conducted in only one country.
I found the information in box 2 to be very important, yet it is presented as an unorganized list of notes. I think a more in-depth description of what organizations and Health IT developers can do to help prevent or at least mitigate the problems you have identified is not only justified, but necessary.	We refer back to Box 2 in the discussion to explain how the results of our study can be used in further research to develop tools to support the design and implementation of health technologies, and to mitigate patient safety unintended consequence. We have also now grouped these recommendations in order to give more coherence to this finding. We also highlight that our participants

	emphasised the importance of embedding user involvement and engagement in technologies' design, a key theme from the information presented in Box 2.
I'd also like to see the authors try to map their work to some sort of framework, model, or theory that could help others to place their findings in context.	We have now added to the discussion more specific comparison with another framework, ISTA, which draws mainly on more specific examples of technologies, highlighting some of the differences and similarities in the findings, as well as more explicitly identifying the novel contribution of our work.

VERSION 2 - REVIEW

**Reviewer**	**1**
**Name**	**Martin, Graham P.**
**Affiliation**	**University of Cambridge**
**Date**	**09-Jul-2024**
**COI**	**None**

This paper uses data from focus groups including patients and members of the public and healthcare staff to identify patient safety risks arising from the introduction of new technologies, along with some possible mitigations. The paper is generally well presented and the findings seem well grounded in the data, and some are quite interesting (e.g. the 'illusion of communication'). I have one major comment, two more minor ones, and a list of specific points that might be addressed to improve presentation and clarity.

My major comment relates to the information provided relating to sampling and recruitment. While the use of a reporting checklist means that a great deal of minor detail is provided, I think some much more important description is missing. In particular, exactly who was recruited and how is not clear from the current text. We are told that "adult patient/public participants with experience of healthcare and who could communicate in English were recruited through our research networks plus an online external research-participant organisation,[25] with purposive sampling to support diversity in age, gender, and ethnicity." I think much more information on these networks (e.g. what kind of people they reached) and on the recruitment organisation is vital, to allow the reader to make a judgement about the representativeness (of whatever kind) of the sample and interpret the findings accordingly. The same is true for the approach to sampling and recruiting healthcare professionals – e.g. how were potential participants identified in the first place?

The reference to the Interactive Sociotechnical Analysis framework on page 14, deep in the discussion, comes as something of a surprise, since it is not (explicitly) referred to as a framing or analytical device for the data until this point. I would suggest that the authors introduce it earlier on, and then use it consistently as they present the data, or perhaps just consider dropping this paragraph, as it does not seem to add much to the paper as currently deployed.

Finally, I would suggest that the passage in the section 'Implications for clinicians and policymakers' is perhaps at risk of over-reaching from one, relatively small, qualitative study. I think it would be premature to use these findings "to develop tools to aid the design and implementation of healthcare technologies" – surely a broader empirical and theoretical basis, and a synthesis of evidence across multiple studies, would provide a more solid foundation for such tools? The similar claim put forward in the abstract could also be reined in.

Minor points:

Page 3 line 30: redundant opening square bracket ([)

Page 5 line 9: could you provide a reference for thematic saturation, and (more importantly) a brief description of how this was operationalised in the study – i.e. what it meant in practice?

Page 5 line 22: the description of ethics approvals reads slightly oddly – was approval gained from two committees? The authors might want to check that this reads as intended

Page 13 line 54: typo: "identified patients by and carers"

Page 14 line 11: typo: "perceive that data presented on technologies to always be correct"

Page 14 line 53: "therefore" – I don't really see how the second part of this sentence follows as a consequence of the first part – consider breaking into separate sentences?

**Reviewer**	**2**
**Name**	**Hamid, Abdullah Al**
**Affiliation**	**King Faisal University**
**Date**	**26-Jul-2024**
**COI**	**None.**

Revision of the manuscript titled: Understanding the unintended patient safety consequences of healthcare technologies: a qualitative study

Authors have done appreciable work, still there is a scope of betterment therefore following are the suggestions/comments which need to be incorporated after which this article may be considered for publication.

1. Although I think it would've been better to include some technology developers in the first place, it is advised to share the outcomes of this study with them to hear their perspectives.

2. How did authors ensure that the online nature of the focus group did not affect the outcomes of the study?.

3. How was the distribution of the included patients decided? Elaborate on the process of participants' recruitment.

4. The results of the abstract included what sounds discussion than results, please revise.

5. Line 36 "engaging with their care" do you mean "carer"?.

6. "Importantly, there has also been little, if any, input from patients, carers, and members of the public into existing tools and frameworks". This statement needs to be supported by evidence from the literature as many of the developers of existing tools claim that they've included all stakeholders.

7. The study design section sounds as theoretical framework, revise it to include more information about study design only please.

8. "HCPs were recruited via email, using a combination of convenience, purposive and snowball sampling", this is a quite interesting sampling method. Could you please explain how did you incorporate all these techniques? Did you consider stratification?

9. "Identifying technology-associated patient safety risks" section is nicely written.

10. It is advised that authors highlight the male/female ratio in the study participants and probably discuss if that could impose any bias or could affect the results.

11. "our findings suggest that technology can increase rather than decrease

this burden, both for patients and HCPs", that's why I believe technology developers should've been included in this study to defend what they principally consider as [the future of healthcare].

12. I suppose it is sometimes more suitable to use passive voice instead of "we" especially when giving some recommendations. The overuse of "we" and "our" in the implication part is examples of what I mean.

VERSION 2 - AUTHOR RESPONSE

COMMENT	RESPONSE
**Reviewer 1 Comments**	
This paper uses data from focus groups including patients and members of the public and healthcare staff to identify patient safety risks arising from the introduction of new technologies, along with some possible mitigations. The paper is generally well presented and the findings seem well grounded in the data, and some are quite interesting (e.g. the 'illusion of communication'). I have one major comment, two more minor ones, and a list of specific points that might be addressed to improve presentation and clarity.	Thank you for your thoughtful review and helpful comments.
My major comment relates to the information provided relating to sampling and recruitment. While the use of a reporting checklist means that a great deal of minor detail is provided, I think some much more important description is missing. In particular, exactly who was recruited and how is not clear from the current text. We are told that "adult patient/public participants with experience of healthcare and who could communicate in English were recruited through our research networks plus an online external research-participant organisation,[25] with purposive sampling to support diversity in age, gender, and ethnicity." I think much more information on these networks (e.g. what kind of people they reached) and on the recruitment organisation is vital, to allow the reader to make a judgement about the representativeness (of whatever kind) of the sample and interpret the findings accordingly. The same is true for the approach to sampling and recruiting healthcare professionals – e.g. how were potential participants identified in the first place?	Thank you for raising this. We have now added more details of the approach we used to sampling and recruitment. The online external research-participant organisation is also referenced for readers who wish for more detailed information on this.
The reference to the Interactive Sociotechnical Analysis framework on page 14, deep in the discussion, comes as something of a surprise, since it is not (explicitly) referred to as a framing or analytical device for the data until this point. I would suggest that the authors introduce it earlier on, and then use it consistently as they present the data, or perhaps just consider dropping this paragraph, as it does not seem to add much to the paper as currently deployed.	The comparison of our findings with Bloomrosen's framework was done *post-hoc*, and so we feel that it would not be appropriate to introduce this in the methods as part of our *a priori* approach. We would prefer to keep this comparison as an important part of comparing

	our findings with the existing literature, but will remove if it the editors recommend this approach. We have however reworded this section of the discussion slightly so that the introduction of this framework (hopefully) does not feel that it comes as a surprise.
Finally, I would suggest that the passage in the section ‘Implications for clinicians and policymakers’ is perhaps at risk of over-reaching from one, relatively small, qualitative study. I think it would be premature to use these findings “to develop tools to aid the design and implementation of healthcare technologies” – surely a broader empirical and theoretical basis, and a synthesis of evidence across multiple studies, would provide a more solid foundation for such tools? The similar claim put forward in the abstract could also be reined in.	Thank you for pointing this out. We have tempered this point both in the main text and in the abstract to avoid over-reaching the findings.
Page 3 line 30: redundant opening square bracket ([)	Thank you, now removed.
Page 5 line 9: could you provide a reference for thematic saturation, and (more importantly) a brief description of how this was operationalised in the study – i.e. what it meant in practice?	A reference has now been added, together with a brief description of how we operationalised this in practice.
Page 5 line 22: the description of ethics approvals reads slightly oddly – was approval gained from two committees? The authors might want to check that this reads as intended	It is a combined research ethics committee which means that the full name is rather long and does indeed read rather oddly! We have now however amended to give the most relevant name for the purposes of this paper in order to avoid confusion.
Page 13 line 54: typo: “identified patients by and carers”	Thank you, amended.
Page 14 line 11: typo: “perceive that data presented on technologies to always be correct”	Thank you, amended.
Page 14 line 53: “therefore” – I don’t really see how the second part of this sentence follows as a consequence of the first part – consider breaking into separate sentences?	Thank you, amended to add clarity as to the point intended.
**Reviewer 2 Comments**	
Authors have done appreciable work, still there is a scope of betterment therefore following are the	Thank you for your helpful suggestions.

suggestions/comments which need to be incorporated after which this article may be considered for publication.	
1. Although I think it would've been better to include some technology developers in the first place, it is advised to share the outcomes of this study with them to hear their perspectives.	Thank you. We will certainly be doing this following publication of the paper.
2. How did authors ensure that the online nature of the focus group did not affect the outcomes of the study?	By the time of this study, in 2021/2022, online meetings were commonplace, and have been extensively used and reported in the literature. They were chosen in this case to increase accessibility for participants who were unable to travel or find the time to attend face to face meetings. As outlined in the paper, we had a dial-in option to support participation by anyone who did not have access to WiFi, network data or a suitable device. Again as outlined in the paper, participants could contribute to the discussion verbally, using the chat function, or using the online Padlet software. We have now added the steps taken to encourage all participants to take part, and that facilitators were experienced with facilitating online meetings and focus groups.
3. How was the distribution of the included patients decided? Elaborate on the process of participants' recruitment.	More details have been added to this section, as also suggested by Reviewer 1.
4. The results of the abstract included what sounds discussion than results, please revise.	We would like to include the text highlighting which results are novel, as this is an important aspect of our findings. The abstract format does not allow for a discussion section to which to move this material. However if the editors feel that it is

	essential to remove this material we can do so.
5. Line 36 “engaging with their care” do you mean “carer”?	We did mean care, rather than carer. We have now reworded this as “healthcare” to avoid any potential ambiguity.
6. “Importantly, there has also been little, if any, input from patients, carers, and members of the public into existing tools and frameworks”. This statement needs to be supported by evidence from the literature as many of the developers of existing tools claim that they’ve included all stakeholders.	We have now added references to support this point.
7. The study design section sounds as theoretical framework, revise it to include more information about study design only please.	We have now reworded the relevant sub-heading to be “Study design and research philosophy” in order to better encapsulate the material presented here
8. “HCPs were recruited via email, using a combination of convenience, purposive and snowball sampling”, this is a quite interesting sampling method. Could you please explain how did you incorporate all these techniques? Did you consider stratification?	More details have been added to this section, as also suggested by Reviewer 1.
9. “Identifying technology-associated patient safety risks” section is nicely written.	Thank you
10. It is advised that authors highlight the male/female ratio in the study participants and probably discuss if that could impose any bias or could affect the results.	We have now added the following statement to the limitations section: “About two thirds of patient/public participants were female; the experiences of male patients may therefore be under-represented”
11. “our findings suggest that technology can increase rather than decrease<br>this burden, both for patients and HCPs”, that's why I believe technology developers should’ve been included in this study to defend what they principally consider as [the future of healthcare].	We felt that we should start by garnering the views of the end users; future research can explore the responses and perspectives of technology developers. We have now added this to the ‘implications for research’ section of the discussion.
12. I suppose it is sometimes more suitable to use passive voice instead of “we” especially when giving some recommendations. The overuse of “we” and	We are not clear of the concern raised by the reviewer here, and believe that the active voice is generally

“our” in the implication part is examples of what I mean.	preferred nowadays. However we are happy to revise the language if required by editorial policy.

VERSION 3 - REVIEW

**Reviewer**	**1**
**Name**	**Martin, Graham P.**
**Affiliation**	**University of Cambridge**
**Date**	**08-Oct-2024**
**COI**	

I feel the authors have adequately addressed the reviewers' critiques. One very minor point - on page 12, the authors use the term "health care", but elsewhere "healthcare" is preferred.
